# Comments on “Dynamic patterns of depression and their association with stroke: insights from CHARLS, ELSA, and HRS”

**DOI:** 10.1097/JS9.0000000000004613

**Published:** 2026-01-23

**Authors:** LinYin Huang, FangJun Wang

**Affiliations:** a The Second School of Clinical Medicine, Zhejiang Chinese Medical University, Hangzhou, China; b Hangzhou Integrative Medicine Hospital Affiliated to Zhejiang Chinese Medical University, Tuina Department , Hangzhou, China

*Dear Editor,*

We read with considerable interest the article by Zhao *et al*, who analyzed trajectories of depressive symptoms and incident stroke across three large ageing cohorts from China, the UK, and the USA^[1]^.By harmonizing CHARLS, ELSA, and HRS, the authors provide important evidence that persistently high or emerging depressive symptoms identify older adults at increased risk of stroke, and they highlight particularly vulnerable subgroups such as women and rural residents. This cross-cohort perspective adds to the growing recognition of depression as a potentially modifiable cerebrovascular risk factor. This correspondence complies with the TITAN 2025 guidelines on transparency, integrity, and responsible use of AI tools in scientific writing (TITAN 2025 Working Group. TITAN 2025 Guidelines: Governing Declaration and Use of AI in Scholarly Publications. 2025. doi: https://doi.org/10.70389/PJS.100082)^[2]^.

We would, however, appreciate further clarification regarding the temporal framing of exposure and outcome. Depressive symptoms were dichotomized at three biennial waves to construct four rule-based trajectories, which were then related to subsequent stroke using logistic regression models^[1]^. If follow-up time after the trajectory window varies substantially between participants or cohorts, odds ratios may conflate short- and long-term risk and be sensitive to differential loss to follow-up. Complementary time-to-event analyses (e.g., Cox models with time from the end of the exposure window to first stroke) could help confirm whether the reported associations are robust when accounting for variable follow-up. Prior work on depression and incident stroke has commonly adopted survival modeling approaches, which may facilitate comparison of effect sizes across studies^[3,4]^.

A second issue concerns the operationalization of depressive trajectories. The four categories (No, Decreasing, Increasing, and Consistently high) are intuitive and clinically appealing. However, dichotomizing CES-D scores at cohort-specific cut-offs and grouping patterns such as “No–Yes–Yes” and “No–No–Yes” within a single “Increasing” category may obscure differences in duration and severity of depressive burden^[1]^. It would be helpful to know whether the authors examined the distribution of continuous CES-D scores within each trajectory, or explored alternative specifications, such as requiring elevation at two or more waves or modeling CES-D as a continuous or ordinal predictor. In the HRS, for example, data-driven latent class approaches have been used to derive depressive symptom trajectories, which were then related to subsequent stroke risk^[4]^. Comparing these latent classes with the present rule-based groups, even descriptively, might strengthen external validity and help clinicians interpret which patterns of symptom persistence are most worrisome.

Finally, a distinctive strength of this study is the inclusion of three nationally representative cohorts with differing sociocultural and health care contexts. The odds ratios for the Consistently high trajectory appear somewhat larger in CHARLS and ELSA than in HRS, although confidence intervals overlap^[1]^. We would be interested to know whether the authors formally tested interaction between cohort and trajectory category and whether they considered a pooled analysis with harmonized covariates to estimate an overall effect and quantify between-cohort heterogeneity. Such analyses could clarify whether the magnitude of risk associated with persistent depressive symptoms is broadly comparable across settings, or whether context-specific factors (e.g., access to mental healthcare or stroke prevention services) modify this association.

In summary, Zhao *et al* provide valuable evidence that dynamic patterns of depressive symptoms carry prognostic information for stroke beyond traditional vascular risk factors^[1]^. Clarifying the temporal framing and measurement of trajectories, and further leveraging the unique multi-cohort design, could help translate these findings into pragmatic screening strategies and targeted interventions for older adults at heightened cerebrovascular risk. We again congratulate the authors on their important contribution and hope that our comments will stimulate further work at the interface between mental health and stroke prevention.

## Data Availability

No datasets were generated or analyzed during the present study.
